# Enhancing the prediction for shunt-dependent hydrocephalus after aneurysmal subarachnoid hemorrhage using a machine learning approach

**DOI:** 10.1007/s10143-023-02114-0

**Published:** 2023-08-19

**Authors:** Dietmar Frey, Adam Hilbert, Anton Früh, Vince Istvan Madai, Tabea Kossen, Julia Kiewitz, Jenny Sommerfeld, Peter Vajkoczy, Meike Unteroberdörster, Esra Zihni, Sophie Charlotte Brune, Stefan Wolf, Nora Franziska Dengler

**Affiliations:** 1 CLAIM – Charité Lab for AI in Medicine, Charité Universitätsmedizin Berlin, Corporate Member of Freie Universität Berlin, Humboldt-Universität Zu Berlin and Berlin Institute of Health, Charitéplatz 1, 10117 Berlin, Germany; 2 Department of Neurosurgery, Charité Universitätsmedizin Berlin, Corporate Member of Freie Universität Berlin, Humboldt-Universität Zu Berlin and Berlin Institute of Health, Charitéplatz 1, 10117 Berlin, Germany; 3 QUEST Centre for Responsible Research, Berlin Institute for Health, Charité Unversitätsmedizin Berlin, Anna-Louisa-Karsch-Str. 2, 10178 Berlin, Germany; 4 School of Computing and Digital Technology, Faculty of Computing, Engineering and the Built Environment, Birmingham City University, 15 Bartholomew Row, Birmingham, B5 5JU UK; 5 Technological University Dublin, Aungier St, Dublin, D02 HW71 Ireland

**Keywords:** Shunt-dependent hydrocephalus, Aneurysmal subarachnoid hemorrhage, Machine learning approach

## Abstract

Early and reliable prediction of shunt-dependent hydrocephalus (SDHC) after aneurysmal subarachnoid hemorrhage (aSAH) may decrease the duration of in-hospital stay and reduce the risk of catheter-associated meningitis. Machine learning (ML) may improve predictions of SDHC in comparison to traditional non-ML methods. ML models were trained for CHESS and SDASH and two combined individual feature sets with clinical, radiographic, and laboratory variables. Seven different algorithms were used including three types of generalized linear models (GLM) as well as a tree boosting (CatBoost) algorithm, a Naive Bayes (NB) classifier, and a multilayer perceptron (MLP) artificial neural net. The discrimination of the area under the curve (AUC) was classified (0.7 ≤ AUC < 0.8, acceptable; 0.8 ≤ AUC < 0.9, excellent; AUC ≥ 0.9, outstanding). Of the 292 patients included with aSAH, 28.8% (*n* = 84) developed SDHC. Non-ML-based prediction of SDHC produced an acceptable performance with AUC values of 0.77 (CHESS) and 0.78 (SDASH). Using combined feature sets with more complex variables included than those incorporated in the scores, the ML models NB and MLP reached excellent performances, with an AUC of 0.80, respectively. After adding the amount of CSF drained within the first 14 days as a late feature to ML-based prediction, excellent performances were reached in the MLP (AUC 0.81), NB (AUC 0.80), and tree boosting model (AUC 0.81). ML models may enable clinicians to reliably predict the risk of SDHC after aSAH based exclusively on admission data. Future ML models may help optimize the management of SDHC in aSAH by avoiding delays in clinical decision-making.

## Introduction

Shunt-dependent hydrocephalus (SDHC) is common after aneurysmal subarachnoid hemorrhage (aSAH) with rates between 7 and 67% [1–5]. Based on different clinical and radiological factors, various scoring systems have been developed to predict the risk of SDHC after aSAH [6, 7]. The two validated scores with the best performance in SDHC prediction are the “Chronic Hydrocephalus Ensuing from SAH Score” (CHESS) and the “Shunt Dependency in aSAH Score” (SDASH) [2, 8, 9].

A recent trend in clinical prediction modelling is the introduction of machine learning (ML) algorithms allowing for the inclusion of a variety of additional complex variables [10, 11]. For example, in aSAH and stroke, such ML algorithms were shown to improve outcome prediction [12–15]. Even though ML-based prediction models have entered nearly all fields of medicine, there is limited evidence on direct comparisons between non-ML and ML-based prediction [13]. ML methods have been criticized for their supposed lack of transparency and confirmability of the impact of variables used, especially in the case of more modern ML techniques, such as deep neuronal networks [25]. Also, there is some debate on which data may be best suited to further enhance the predictive capabilities of ML models. For example, given that any prediction of an event should be done as early as possible in order to adjust care decisions and potentially influence outcomes, there is an inherent need to feed ML models with early variables, such as admission data, rather than variables from later stages of clinical management, such as, in the case of SDHC, the amount of cerebrospinal fluid (CSF) drained during the first 14 days after aSAH [16, 17].

We therefore performed a study to compare the performance of the non-ML-based scores CHESS and SDASH to different ML models in predicting SDHC after aSAH. We also compared various ML models among each other, some relying exclusively on variables available on admission, others adding the amount of 14-day CSF volumes as a late variable.

## Methods and materials

### Patient management and data collection

We retrospectively evaluated prospectively collected clinical, radiographic, and laboratory data of 408 consecutive patients hospitalized with aSAH at our department between January 1, 2009, and December 31, 2015. Local ethics committee approval was obtained (EA1/291/14). The only inclusion criterion was aSAH confirmed by CT or xanthochromic cerebrospinal fluid on admission. If the hemorrhage was due to trauma or we were not able to identify an aneurysmal source of the bleeding, patients were excluded. The clinical condition was assessed based on the Hunt & Hess grading system [18]. Radiographic parameters were assessed based on the admission CT. Semi-quantitative radiographic grading of the thickness of the subarachnoidal blood clot was performed according to the BNI grading system [19]. Other radiographic parameters on CT were as follows: the presence of intraventricular hemorrhage (IVH), intracerebral hemorrhage (ICH), early infarction (EI), posterior location of the aneurysm (post. loc. AY), and acute hydrocephalus (aHP). aHP was defined according to Bae and colleagues based on third ventricle enlargement and periventricular low density on CT within 72 h of the aSAH, combined with mental deterioration or impaired consciousness or memory, gait disturbance, and urinary incontinence [20]. The following laboratory serum parameters were assessed on admission: creatinine in mg/dl, glucose in mg/dl, and C-reactive protein (CRP) in mg/l.

For aSAH management, early aneurysm occlusion was attempted within 48 h after aSAH, according to previously published guidelines [21, 22]. For aHP management, the standard protocol established by Jabbarli and colleagues was followed [9]. In the acute phase, an external ventricular drain (EVD) or lumbar drain (LD) was placed. At a later stage, a ventriculoperitoneal shunt was placed if patients could not be weaned off the external ventricular drain (EVD) or lumbar drain (LD) within 14 days.

### Outcome assessment

Shunt dependency was examined in patients that survived the index hospital stay. It was assessed based on patient files from routine control visits 6–12 months after aSAH. If no routine follow-up data was available, data were obtained by telephone interview.

### Scores

For the assessment of the CHESS score to predict SDHC, the following variables were included: Hunt & Hess grade ≥ 4 (1 point), aneurysm location in the posterior circulation (1 point), aHP (4 points), IVH (1 point), and early cerebral infarction on CT (1 point) [9]. The BNI score was established according to the thickness of the subarachnoid blood clot perpendicular to a cistern or fissure (1: no visible SAH; 2: ≤ 5 mm; 3: 6–10 mm; 4: 11–15 mm; 5: 16–20 mm) [19]. The SDASH ranges from 1 to 4 points and was calculated as recently described [2]. It includes the following variables: presence of aHP (2 points), BNI score ≥ 3 (1 point), and Hunt & Hess grade ≥ 4 (1 point). SDASH and CHESS scoring systems were compared using a conventional area under the curve (AUC) calculation as previously described [2].

### Feature selection

**Fig. 1:**
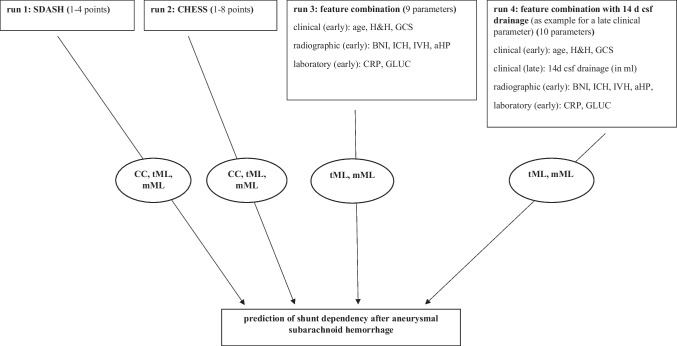
Prediction paradigms for shunt dependency after subarachnoid hemorrhage. Abbreviations: aHP, presence of acute hydrocephalus; AY, aneurysm; BNI, Barrow Neurological Institute scale for the thickness of subarachnoid hemorrhage; CC, conventional calculation; CRP, C-reactive protein; csf, cerebrospinal fluid; H&H, Hunt & Hess scale; ICH, intracerebral hemorrhage; IVH, intraventricular hemorrhage; tML, traditional machine learning with generalized linear models; Least absolute shrinkage and selection operator regression (LASSO) and ElasticNET; mML, modern machine learning with tree boosting (CatBoost), Naive Bayes (NV), and multilayer perceptron (MLP) neuronal network models

Among 408 patients with aSAH in the data base, 116 patients had to be excluded as they did not survive the initial phase of the disease or based on missing information on SDHC. We therefore included 292 patients in this study. Among those, only a few variables were missing (age: 0.7%, ICH: 0.3%, aneurysm location: 0.8%). Mean/mode imputation was used in each fold to impute missing values (see section “Model training and validation”). Input features were included if a ratio of at least 1 to 4 for binary variables (absence/presence) was reached. Features that were shown to be associated with SDHC were included (Table 1). This resulted in the following variables being used in the analyses: age, Hunt & Hess grade, BNI grade, presence of aHP, presence of ICH, presence of IVH, serum levels of CRP, and glucose on admission. As a further feature, we used the amount of cerebrospinal fluid (CSF) drainage (in ml) over the first 14 days after aSAH, as it is an established risk factor of SDHC after aSAH and as it represents a “late feature” in a separate run (Fig. 1) [8, 23]. Categorical features with at least three categories were transformed into binary features if they had too few instances per category. The following dichotomizations were used: radiologically defined ICH “yes”/ “no,” and aneurysm location in the anterior circulation “yes”/ “no.” Thus, all variables were either binary or continuous.

**Table 1:** Patient characteristics

	Total study population (*n* = 292)	SDHC present (*n* = 84)	No SDHC present (*n* = 208)	*p*-value
Age in years, median [IQR]	53 [45–61]	84 [58–64]	51 [45–59]	**0.01**
Female sex (*n*)	68.2% (199)	70.1% (59)	67.3% (140)	0.627
GCS, median [IQR]	15 [8–15]	9 [3–14]	15 [13–15]	** < 0.01**
Hunt & Hess score (*n*)	-	-	-	** < 0.01**
I	30.8% (90)	9.5% (8)	39.4% (82)	
II	23.3% (68)	20.2% (17)	24.5% (51)	
III	17.8% (52)	17.9% (15)	17.8% (37)	
IV	12.2% (35)	22.6% (19)	7.7% (16)	
V	16.1% (47)	29.8% (25)	10.6% (22)	
Sum CFS drainage within 14 days, median [IQR]	1303 [576–2093]	1879 [1319–2391]	847 [336–1786]	** < 0.01**
Presence of acute hydrocephalus (*n*)	33.6% (98)	63.1% (51)	21.6% (45)	** < 0.01**
Presence of intraventricular hemorrhage (*n*)	47.3% (138)	75.0% (63)	36.1% (75)	** < 0.01**
Presence of intracerebral bleeding (*n*)	25.7% (75)	38.1% (32)	20.7% (43)	**0.02**
Early infarction (*n*)	11.0% (32)	11.9% (10)	10.6% (22)	0.748
BNI score (*n*)				** < 0.01**
1	8.2% (24)	2.4% (2)	10.6% (22)	
2	18.5% (54)	7.1% (6)	23.1% (48)	
3	31.8% (93)	34.5% (29)	30.8% (64)	
4	31.8% (93)	39.3% (33)	28.8% (60)	
5	9.6% (28)	16.7% (14)	6.7% (14)	
Localization of Ay (*n*)				0.325
MCA	27.4% (80)	23.8% (20)	28.8% (60)	
ACA	36% (105)	38.1% (32)	25.1% (73)	
ICA	22.6% (66)	19.0% (16)	24.0% (50)	
Posterior circulation	14.0% (41)	19.0% (16)	12.0% (25)	
Size of Ay, mm, median [IQR]	6% [4–8]	7 [5–10]	6 [4–8]	0.078
CRP, median [IQR]	1.0 [0.3–3.1]	1.8 [0.5–5.9]	0.8 [0.3–2.4]	**0.006**
Creatinine, median [IQR]	0.7 [0.6–0.8]	0.7 [0.6–0.9]	0.7 [0.6–0.8]	0.484
Glucose, median [IQR]	138 [114–117]	158 [127–202]	132 [111–153]	** < 0.01**

Data is presented in % (*n*) or median and interquartile range. *p*-values were determined via the Mann–Whitney *U* test and the chi-quadrat Pearson test. Abbreviations: *AY*, aneurysm; *CRP*, C-reactive protein; *CSF*, cerebrospinal fluid; *IQR*, interquartile ratio; *MCA*, middle cerebral artery; *ACA*, anterior cerebral artery; *ICA*, internal carotid artery. Creatinine and glucose are presented in mg/dl and C-reactive protein (CRP) in mg/l

### Model selection

Additional to the conventional area under the receiver operating characteristic curve calculation, ML models were trained for each score (run 1, CHESS; run 2, SDASH). A combined model with a set (run 3) of individual features available on admission was designed to include all clinically relevant (age, GCS) and laboratory parameters (glucose, CRP) as well as radiographically important parameters (aHP, IVH, ICH) on admission independent from SDASH and CHESS model calculation was trained. The additional run 4 included the features of run 3 and the amount of CSF drained during the first 14 days after aSAH (Fig. 1, Table 1).

### Machine learning framework

To train ML models, a publicly available ML framework for predictive modelling was used utilizing standard ML libraries in Python. Its code can be accessed on GitHub (https://github.com/prediction2020/explainable-predictive-models), and details on the technical implementation can be found in previous open-access publications [14, 24]. A supervised ML approach was trained on all clinical parameters and scores listed in Table 1 to predict SDHC. Our dataset contained 84 positive (SDHC present) and 208 negative (no SDHC present) cases. This provided a reasonably balanced dataset and thus refrained from a sub-sampling approach that would have limited the amount of data for model training.

### Applied algorithms

**Fig. 2:**
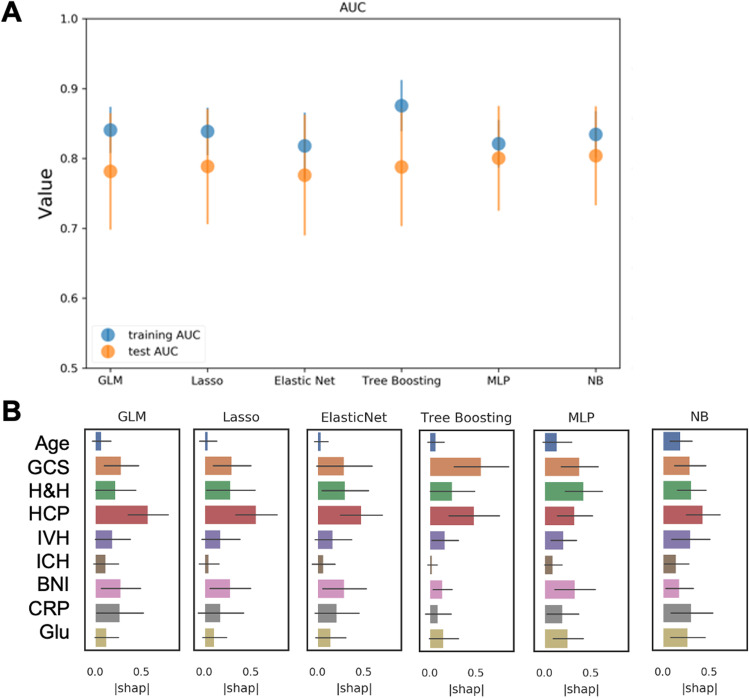
Performance and feature rating for the combined feature set for features available early in the clinical course of aneurysmal subarachnoid hemorrhage (run 3). **A** GLM, LASSO, and tree boosting (CatBoost) models reached the highest AUC in training (0.84/0.88) and MLP and NB in the test (0.80, respectively). A large difference between training and test was seen in tree boosting which may be an indication of overfitting. **B** GCS, Hunt & Hess, and the presence of early hydrocephalus were defined as the most important factors in the majority of models. Abbreviations: aHP, presence of acute hydrocephalus; BNI, Barrow Neurological Institute scale for thickness of subarachnoid hemorrhage; CRP, C-reactive protein early; GLM, generalized linear model; HCP, presence of early hydrocephalus; H&H, Hunt & Hess scale; ICH, intracerebral hemorrhage; IVH, intraventricular hemorrhage; MLP, multilayer perceptron; NB, Naive Bayes

**Fig. 3:**
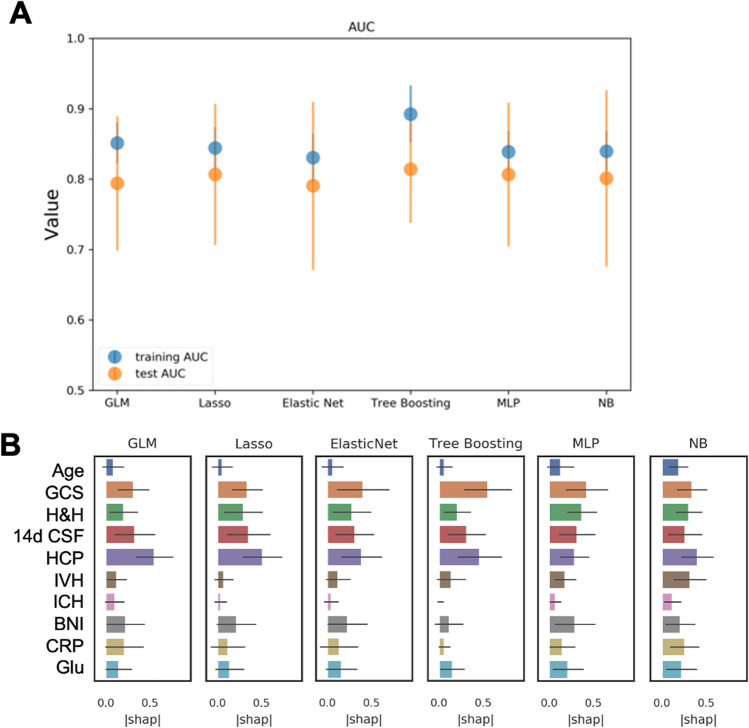
Performance and feature rating for the combined feature set including 14-day cerebrospinal fluid drainage *(*run 4). **A** The tree boosting (CatBoost) model reached the highest AUC in training (0.89) and so did MLP and tree boosting in the test (0.81, respectively). Also, a larger difference between the training and test set in the tree boosting model was noted which may be indicative of overfitting. **B** The presence of early hydrocephalus, GCS, Hunt & Hess, and the amount of cerebrospinal fluid drainage within the first 14 days after the hemorrhage were defined as the most important factors in the majority of models. IVH, BNI, creatinine, and glucose were rated next important with varying extent throughout the models. Abbreviations: aHP, presence of acute hydrocephalus; BNI, Barrow Neurological Institute scale for thickness of subarachnoid hemorrhage; CRP, C-reactive protein; 14d CSF, the volume of drained cerebrospinal fluid within the first 14 days after the hemorrhage; GLM, generalized linear model; HCP, presence of early hydrocephalus; H&H, Hunt & Hess scale; ICH, intracerebral hemorrhage; IVH, intraventricular hemorrhage; MLP, multilayer perceptron; NB, Naive Bayes

We used six different algorithms for all RUN selections. Three types of generalized linear models (GLM) represented traditional ML models: a plain GLM, an L1 regularized GLM (equivalent to LASSO logistic regression—LASSO), and a GLM elastic net with an additional L2 regularization (ElasticNET). We also included more modern ML models like a tree boosting algorithm (CatBoost), a Naive Bayes (NB) classifier, and a type of artificial neural network, the multilayer perceptron (MLP). For run 3 (“feature combination”) and run 4 (“feature combination with 14-day CSF drainage”), feature importance ratings were calculated for all algorithms using SHapley Additive exPlanations (SHAP) values (Table 2, Figs. 2 and 3). To minimize potential confounding effects on predictive performance, the variance inflation factor (VIF) was applied to assess multicollinearity for all features [25].

**Table 2:** Predictive performance of scores and machine learning models in training and test

Run	CC	GLM	GLM_LASSO	GLM_ElasticNET	MLP	NB	Tree boosting (CatBoost)
SDASH (run 1)	0.78	**0.77** (0.09) 0.78 (0.04)	**0.76** (0.09) 0.78 (0.04)	**0.74** (0.15) 0.77 (0.10)	**0.77** (0.09) 0.78 (0.05)	**0.77** (0.09) 0.78 (0.04)	**0.77** (0.10) 0.78 (0.05)
CHESS (run 2)	0.77	**0.77** (0.08) 0.78 (0.03)	**0.76** (0.08) 0.78 (0.03)	**0.76** (0.14) 0.78 (0.04)	**0.76** (0.08) 0.78 (0.04)	**0.77** (0.08) 0.78 (0.03)	**0.76** (0.07) 0.78 (0.03)
Feature combination (age, GCS, HH, HCP, IVH, ICH, BNI, CRP, Gluc) (run 3)		**0.78** (0.08) 0.83 (0.03)	**0.79** (0.08) 0.84 (0.03)	**0.78** (0.09) 0.82 (0.05)	**0.80** (0.07) 0.82 (0.03)	**0.80** (0.07) 0.83 (0.03)	**0.79** (0.08) 0.88 (0.04)
Feature combination with 14-day CSF drainage (age, GCS, H&H, CSF drainage in 14 days, HCP, IVH, ICH, BNI, CRP, Gluc) (run 4)		**0.79** (0.10) 0.85 (0.03)	**0.81** (0.10) 0.84 (0.03)	**0.79** (0.12) 0.83 (0.03)	**0.81** (0.10) 0.84 (0.03)	**0.80** (0.13) 0.84 (0.03)	**0.81** (0.08) 0.89 (0.04)

The area under the curve for the respective training (below) and test (bold, above) set is depicted for each model trained. In column 2, we present the results of a conventional calculation (CC) of the area under the curve for SDASH and CHESS. Columns 3 to 5 on the left represent traditional machine learning (ML) models and the right three columns contain more modern machine learning techniques like MLP (multilayer perceptron), NB (Naive Bayes), and a tree boosting algorithm called CatBoost

Abbreviations: *aHP*, presence of acute hydrocephalus; *BNI*, Barrow Neurological Institute scale for the thickness of subarachnoid hemorrhage; *CC*, conventional calculation of predictive power for scores; *CHESS*, Chronic Hydrocephalus Ensuing from SAH Score; *CRP*, C-reactive protein; *csf*, cerebrospinal fluid; *H&H*, Hunt & Hess scale; *Gluc*, glucose; *ICH*, intracerebral hemorrhage; *IVH*, intraventricular hemorrhage; *ML*, machine learning; *SDASH*, Shunt Dependency in aSAH Score

### Model training and validation

All data were split randomly into training and test sets at a 4:1 ratio. On both sets, we performed mean/mode imputation and feature scaling using zero-mean unit variance normalization based on the training set. A tenfold cross-validation was used for hyperparameter tuning. This process was repeated in 50 shuffles.

### Performance assessment

Receiver operating characteristic (ROC) analysis was used to test the model performance on the test set by measuring the AUC. Performance was also assessed based on accuracy, average class accuracy, precision, recall, F1 score, negative predictive value, and specificity. The Brier score was used to quantify model calibration. According to Hosmer and Lemeshow, we used the following classification system for AUC: 0.7 ≤ AUC < 0.8, “acceptable”; 0.8 ≤ AUC < 0.9, “excellent”; AUC ≥ 0.9, “outstanding” [26, 27].

### Interpretability assessment

To facilitate the comparability of feature ratings across models, we scaled the absolute values of the SHAP feature importance scores to the unit norm and, for each of the 50 shuffles, rescaled them to a range from 0 to 1 with their sum equal to 1. Each features’ means and standard deviations, calculated on the test sets over all shuffles, were reported as the final rating measures.

## Results

### Patient characteristics

Two hundred ninety-two patients with a median age of 53 years [IQR 45; 61] and a female to male ratio of 2:1 were included in the analysis. Of these, 28.8% (*n* = 84) developed SDHC. Clinical, radiographic, and laboratory data in the entire patient cohort are depicted in Table 1.

### SDASH and CHESS score validity in conventional and ML-based outcome prediction

A conventional AUC calculation without ML algorithms revealed an AUC of 0.77 for CHESS and 0.78 for SDASH. To maintain comparability of scores in the ML-based models, CHESS and SDASH score values were introduced to the ML framework. Here, both scores reached acceptable prediction in training (AUC range: 0.77–0.78) and test (AUC range: 0.74–0.77) without remarkable differences between classic ML models (GLM, LASSO, ElasticNET) and more modern ML models (MLP, NB, CatBoost) (Table 2).

### ML-based prediction with early parameters

The feature combination set with variables available on admission (run 3) revealed excellent predictive performance in training (AUC range: 0.82–0.88 [tree boost]) and reached acceptable performances in the GLM models and tree boosting model in the test set (AUC range: 0.78–0.79). In the test set, excellent performance was reached for the MLP and NB model (AUC 0.80, respectively (Table 2, Fig. 2A). The most important features were aHP, GCS, or Hunt & Hess H, and to varying extents in different models, BNI, IVH, CRP, and glucose (Fig. 2B).

### ML-based prediction with early parameters and CSF volume drained within 14 days

The addition of the volume of CSF drained within the first 14 days as a late parameter after aSAH revealed further increases of the AUC to excellent performances in the test for the GLM (0.81), MLP (0.81), NB (0.80), and tree boosting (0.81) model (Table 2, Fig. 3A). When CSF was added to run 4, this variable gained importance being rated second or third highest after GCS or Hunt & Hess and HCP in most models (Fig. 3B).

## Discussion

We present the first study on the prediction of SDHC after aSAH comparing traditional scoring systems to ML-based models and different ML-based models among each other. The main result of this analysis is that predictive performances of conventional scores SDASH and CHESS were reproduced with an ML-based analysis of these scores. A combination of features that were available already at admission and that were shown to be relevant for SDHC was introduced to an ML model approach, which resulted in an increase of predictive performance to an AUC of 0.80, which represents excellent prediction performance. Adding the late variable “14-day CSF volume” further improved the prediction of SDHC.

The fact that, in our study, the examined ML-based calculation of SDASH and CHESS scores reached predictive performances comparable to traditional and validated SDASH and CHESS scores suggests that ML approaches are valid and reproducible in predicting SDHC after aSAH [2, 8, 9]. This is of particular importance, as ML methods have previously been labeled “black-box-models” [28]. Especially more modern ML models, e.g., deep neural networks, have been criticized for being designed to identify and make use of associations between features rather than describe those associations in detail, which may limit the interpretability of the results [29, 30]. ML methods are therefore still predominantly used in the domain of predicting outcomes and complications, where their application is considered safe enough to allow them to learn continuously, as standard care is not affected while model updates can be carried out [31]. As ML models learn, it is important to identify the point at which their predictive performance surpasses validated non-ML prediction models. To this end, in our view, a continuous direct comparison between ML output and corresponding non-ML results is needed to prevent ML models from being perceived as black boxes simply producing different levels of AUC values. Critics of ML models in outcome prediction stress the lack of transparency as shown in a recent study [32]. Currently unanswered questions such as “What level of model transparency is required?” and “Do we understand the model outputs and whether they are unreliable and therefore not to be trusted?” may influence the future clinical utility of ML-based applications [32, 33]. This prompted us to compare ML outputs to well-established standards that are currently used in clinical practice. Moreover, our study specifically aims to identify and document factors contributing to potentially superior outputs of ML models and may therefore serve as a basis for future research.

It is somewhat surprising that this step of ML model validation is rarely done. A strength of the presented analysis is that it allows for a direct comparison of established, validated score calculations with traditional and more modern ML methods, which, in the case of predicting SDHC after aSAH, has never been done before. Only one other study exists examining ML methods in predicting SDHC after aSAH, albeit without comparison to non-ML methods [29]. In that study, on the basis of 368 patients and 32 variables, various ML algorithms were used, and the highest performance was reached using a distributed random forest model based on 21 variables, leading to a predictive performance with an AUC of 0.88 in validation and 0.85 in test with sixfold cross-validation [34]. That model included clinical and radiographic variables available on admission but also late variables, such as type of aneurysm treatment, ICU stay, time from symptom onset to treatment initiation, presence of fever, meningitis, treatment complications, and other infections. In our study, the ML-based feature combination (run 3) was solely based on features available at admission but also reached excellent prediction with the MLP and NB model.

Given that the prediction of any event becomes more valuable the sooner it is made, our finding that an ML algorithm based exclusively on admission data can predict SDHC with excellent performance is encouraging [17]. Of note, we did in fact observe a mild increase in predictive performance once the amount of 14-day CSF drainage was added to ML algorithms. In discussing the merits of late versus early variables, it is important to note that it hardly surprises that, in the case of our study, the late variable “14-day CSF volumes” led to improved prediction of SDHC, since this feature merely quantifies the failed attempt to wean patients off of EVD or LD, which is basically a symptom of SDHC rather than a risk factor. It is important to stress that any delay, in the case of our analysis a delay of 14 days in order to assess total CSF drainage, may outweigh the benefits of improved predictive performance, since it can increase the risk of complications. For example, delayed prediction of SDHC after aSAH may mean extended duration of external ventricular catheterization, which has been shown to be associated with increased rates of catheter-associated infection [35]. The finding that later variables may, to a certain degree, improve ML performance is in line with evidence from a recent study on ML-based prediction of discharge outcomes after aSAH, which describes the improvement of prediction once features from later phases of in-hospital stay are added to ML algorithms [16]. However, in that study, as well, the predictive performance of ML models including later features was not substantially better than ML algorithms based exclusively on admission data. In the case of SDHC prediction in aSAH, later variables that were shown to be associated with SDHC in non-ML prediction models were rebleeding and in-hospital complications such as meningitis, pneumonia, vasospasm, and ischemic stroke [3–7]. Whether the inclusion of these additional late factors may have improved our model remains elusive.

Another finding in our study was that ML models using only CHESS or SDASH data in the prediction of SDHC showed inferior performance when compared with ML models using variables more complex and comprehensive than CHESS or SDASH, such as the semi-quantitative measure BNI for the thickness of subarachnoidal blood, the presence of intracerebral hemorrhage, early infarction, and the age and laboratory serum parameters that were included. These additional factors were chosen, because their predictive value for SDHC was established in previous studies [2, 6, 8]. Our more multilayered ML models resulted in prediction improvements by 0.01 to 0.04 AUC points compared to CHESS and SDASH. It is reasonable to assume that future ML models with more multi-layered sets of admission features may further enhance predictive performances on admission. This could significantly influence treatment strategies, such as the timing of implantation of a ventriculoperitoneal shunt, or transfer to intensive care. Whether the addition of radiographic source data or other parameters previously shown to be associated with SDHC, e.g., cerebrospinal fluid markers such as total protein, red blood cell count, interleukin-6, or glucose, would have improved the predictive performance of the models used in our study remains to be tested [36]. The same goes for later variables previously shown to be associated with SDHC in non-ML prediction models, such as rebleeding and in-hospital complications such as meningitis, pneumonia, vasospasm, and ischemic stroke [37–41]. Whether the inclusion of such later factors would have impacted our models’ predictions is uncertain. Our data suggest that ML methods are suited for testing this, with more modern ML models like tree boosting, NB, and the MLP model generating better predictions, especially for the mixed parameter sets in run 3 and run 4.

The main strength of our study is the maintenance of transparency and comparability to existing models as well as between the different models, as our current approach examined established scoring systems with established statistical models in comparison to ML techniques. Nevertheless, the following limitations deserve to be mentioned. Our study was conducted at a single institution, which may limit the generalizability of our findings. Also, the retrospective and non-randomized nature of the analysis may introduce some selection bias and does not allow for causational interpretation of our results. Given the exclusion of non-survivors from our analysis, a bias towards patients with comparably good Hunt & Hess grades cannot be ruled out. However, this method is in line with previous studies on predictive factors for SDHC in aSAH and allows for comparability of our results to those reports [1–5]. A factor that may decrease comparability to other studies are variations in local treatment strategies concerning the timing or even the necessity to place a shunt after surgery. One may argue that true objective cut-offs for whether or when best to implant a shunt system do not exist. In our study, we adhered to an established protocol, as mentioned previously [2, 9]. However, the retrospective nature of our study has limits in terms of adherence to this protocol which we did not examine within this analysis. Given that shunt rates described in the literature range widely between 7 and 67%, our rate of 28.8% reflects a reasonable value, which is comparable to shunt rates published in studies using the same protocol [1–5, 9].

The fact that, in our study, the predictive capabilities of ML methods were comparable or better to the standard scores SDASH and CHESS suggests that future continual learning could one day enable the ML models to outmatch SDASH and CHESS. Once the superiority of ML prediction in SDHC after aSAH is reached, a randomized controlled trial will be necessary to validate these findings, before ML methods can become standard tools optimizing SDHC prediction in real time.

### Conclusions

Our study is the first to present comparative data for SDHC prediction after aSAH for validated scores and state-of-the-art ML techniques. It suggests that ML models may enable clinicians to reliably predict the risk of SDHC based exclusively on admission data. Since early prediction of SDHC is key in aSAH management, future ML models could help optimize care for SDHC in aSAH by avoiding delays in clinical decision-making.

## Data Availability

Data is available upon reasonable request.
